# Primary Cilia Negatively Regulate Melanogenesis in Melanocytes and Pigmentation in a Human Skin Model

**DOI:** 10.1371/journal.pone.0168025

**Published:** 2016-12-12

**Authors:** Hyunjung Choi, Ji Hyun Shin, Eun Sung Kim, So Jung Park, Il-Hong Bae, Yoon Kyung Jo, In Young Jeong, Hyoung-June Kim, Youngjin Lee, Hea Chul Park, Hong Bae Jeon, Ki Woo Kim, Tae Ryong Lee, Dong-Hyung Cho

**Affiliations:** 1 Department of Gerontology, Graduate School of East-West Medical Science, Kyung Hee University, Yongin, Gyeonggi-do, Republic of Korea; 2 R&D Unit, AmorePacific Corporation, Yongin, Gyeonggi-do, Republic of Korea; 3 Department of Medical Science, Korea University Ansan Hospital, Ansan, Gyeonggi-do, Republic of Korea; 4 Biomedical Research Institute, MEDIPOST Corporation, Seongnam, Gyeonggi-do, Republic of Korea; 5 Department of Pharmacology, Wonju College of Medicine, Yonsei University, Wonju, Gangwon-do, Republic of Korea; Sungkyunkwan University, REPUBLIC OF KOREA

## Abstract

The primary cilium is an organelle protruding from the cell body that senses external stimuli including chemical, mechanical, light, osmotic, fluid flow, and gravitational signals. Skin is always exposed to the external environment and responds to external stimuli. Therefore, it is possible that primary cilia have an important role in skin. Ciliogenesis was reported to be involved in developmental processes in skin, such as keratinocyte differentiation and hair formation. However, the relation between skin pigmentation and primary cilia is largely unknown. Here, we observed that increased melanogenesis in melanocytes treated with a melanogenic inducer was inhibited by a ciliogenesis inducer, cytochalasin D, and serum-free culture. However, these inhibitory effects disappeared in GLI2 knockdown cells. In addition, activation of sonic hedgehog (SHH)-smoothened (Smo) signaling pathway by a Smo agonist, SAG inhibited melanin synthesis in melanocytes and pigmentation in a human skin model. On the contrary, an inhibitor of primary cilium formation, ciliobrevin A1, activated melanogenesis in melanocytes. These results suggest that skin pigmentation may be regulated partly by the induction of ciliogenesis through Smo-GLI2 signaling.

## Introduction

The primary cilium is a major cellular sensory organelle that functions as antennae for sensing extracellular information in many cell types [1]. Interactions of cells and external stimuli including chemical, mechanical, and paracrine signals are mediated by the primary cilium [2]. Furthermore, recent evidence has suggested that the primary cilium also plays an important role in tumorigenesis and embryonic development [3,4]. Aberrant ciliogenesis is associated with human diseases, known as ciliopathies, including Bardet–Biedl syndrome, neurosensory impairment, renal polycystic diseases, diabetes, and hypertension [5–7]. Cilia are microtubule-based cellular organelles that are maintained by an intraflagellar transport (IFT) mechanism. IFT is required for the elongation of the cilia, and is a major intracellular transport system that carries non-membrane-bound particles and building materials from the cell body to the growing cilia [8,9]. The cilia membrane includes a number of receptor proteins, ion-conducting channels, and signal transduction components [2]. Primary cilium formation is regulated by SHH and Wnt signaling [10,11]. In the SHH signaling pathway, SHH ligands bind to its receptor and the binding activates smoothened (Smo) protein by phosphorylation. The phosphorylated Smo protein in turn dissociates GLI proteins from repressors, kinesin superfamily 7 (Kif7), and suppressor of fused (SUFU). Finally, the detached GLI protein translocates into the nucleus to turn on SHH target genes [12]. Therefore, primary cilium formation is activated by the SHH signaling pathway. In addition, as a signaling hub, the Wnt signaling pathway regulates primary cilium formation [13]. Cells without cilia enhance Wnt signaling, whereas cells with multiple cilia reduce its responses [14]. The pigmentation of human skin is influenced by external stimuli such as ultraviolet radiation (UVR), microenvironmental stiffness, inflammation, and hormones [15,16]. The pigmentation of human skin is regulated by a complex process involving the synthesis and distribution of melanin. Melanin is synthesized in melanosomes, which are synthesized within melanocytes, transported to the dendrite tips of melanocytes, and then transferred to keratinocytes. Synthesis of melanin is regulated mainly by alpha-melanocyte stimulating hormones (α-MSH), which bind to the melanocortin 1 receptor (MC-1R) and increase melanin synthesis through activation of the cAMP pathway [17]. cAMP production results in the activation of its downstream molecules protein kinase A (PKA) and cAMP-responsive element binding (CREB) protein 1. Activation of these proteins up-regulates the expression of microphthalmia-associated transcription factor (MITF) [18]. MITF is a master regulator of melanogenesis that controls the expression of melanogenic enzymes, such as tyrosinase, TRP-1, and TRP-2 [18] and melanocyte development [19].

It was previously reported that CREB-binding protein (CBP) is a transcriptional coactivator of GLI, and the MITF and GLI2 genes were found to be inversely expressed in various melanoma cell lines [20]. In melanoma cells, primary cilia are reduced compared with melanocytes in nevi [21]. The primary cilia also have an important role in skin development. Differentiation of keratinocytes is suppressed by ciliary mutants and the elimination of cilia induces hyperproliferation of keratinocytes [22]. Hair follicle morphogenesis is initiated by signaling through primary cilia [23]. However, the functions of primary cilia in melanocytes of skin have not been determined.

In this study, we addressed the effect of ciliogenesis on melanogenesis. We found that the induction of primary cilium formation by Smo-GLI2 signaling suppresses melanin production by reducing the expression of melanogenic enzymes. However, the suppression of primary cilium formation by ciliobrevin A1 induced melanin synthesis. These effects of primary cilia on pigmentation were additionally confirmed in a human skin model. Overall, our data suggest that ciliogenesis has a critical function in melanogenesis.

## Materials and Methods

### Cell culture

Normal human epidermal melanocytes (NHEMs) from the neonatal foreskin of moderately or darkly pigmented donors were purchased from Cascade Biologics (Portland, OR). The Melan-a melanocyte cell line, which is an immortalized mouse melanocyte cell line derived from a C57BL/6 J (black, a/a) mouse, was kindly provided by Dr. Dorothy C. Bennett (St. George's Hospital Medical School, London, UK). The B16F1 melanoma cell line was purchased from ATCC (Manassas, VA). Normal human melanocytes were maintained in M-254 medium with Human Melanocyte Growth Supplements (HMGS) (Cascade Biologics, Inc., Mansfield, UK). The Melan-a cells were grown in RPMI 1640 medium supplemented with 10% (v/v) fetal bovine serum, 1% (v/v) penicillin-streptomycin, and 0.2 μM phorbol 12-myristate 13-acetate. The B16F1 mouse melanoma cell lines were cultured in DMEM supplemented with 10% (v/v) fetal bovine serum and 1% (v/v) penicillin-streptomycin. Three-dimensional human skin substitute (MelanoDerm^™^, MEL-312-B) was purchased and maintained in EPI-100-NMM-113 media optimized with KFG, β-FGF, and α-MSH according to the manufacturer’s instructions (MatTek, Seoul, Korea). The change in lightness (ΔL) value of the skin model was calculated from the L value (lightness index) measured by a colorimeter (CR-300; Konica Minolta, Tokyo, Japan), as follows: ΔL = L (treatment skin) − L (control skin) at 13 and 20 days. An increase in the ΔL value indicates the chemical-induced hypopigmentation of the skin [24].

### Reagents and small interfering RNA transfection

Cytochalasin D, ciliobrevin A1, α-MSH, arbutin, and kojic acid were purchased from Sigma-Aldrich (St. Louis, MO). The Smo-agonist SAG was purchased from Calbiochem (San Diego, CA). Previously validated siRNAs targeting mouse GLI2 #1 (5ʹ-CCAACCAGAACAAGCAGAACA-3ʹ) and #2 (5ʹ-GAACACGAAGGCTGTAACAAA-3ʹ) [25] and a negative control siRNA (5ʹ-CCUACGCCACCAAUUUCGU-3ʹ) were synthesized by Genolution (Seoul, Korea). Melan-a and B16F1 cells cultured in 60 mm dishes were transfected with siRNA using Lipofectamine^®^ RNAi MAX (Invitrogen, Carlsbad, CA) according to the manufacturer’s instructions. After incubation with siRNA for 24 h, the cells were treated with each chemical for 48 h.

### Melanin assay

Melanin contents in melanocytes were determined as described previously [26]. Cells were harvested with trypsin/EDTA, centrifuged for 5 min at 1000 × *g*, washed with PBS twice, and then final cell pellets in tubes were photographed. Cell pellets were dissolved with 1 N NaOH. The homogenized cell extracts were transferred into 96-well plate in triplicate and relative melanin content was determined by measuring absorbance at 405 nm using an ELISA plate reader.

### Western blot analysis

All lysates were prepared in 2 × Laemmli sample buffer (62.5 mM Tris-HCl, pH 6.8, 25% [v/v] glycerol, 2% [w/v] SDS, 5% [v/v] β-mercaptoethanol, and 0.01% [w/v] bromophenol blue) (Bio-Rad, Hercules, CA). All cellular proteins were quantified using Bradford solution (Bio-Rad) according to the manufacturer’s instructions. The samples were then separated by SDS-PAGE and transferred to a PVDF membrane (Bio-Rad). After blocking with 4% (w/v) skim milk in TBST (25 mM Tris, 140 mM NaCl, and 0.05% [v/v] Tween^®^ 20), the membranes were incubated overnight with specific primary antibodies. Anti-polyglutamylated-tubulin antibody (AG-20B-0020, 1:1000) was purchased from Adipogene (San Diego, CA); anti-GLI2 antibody (18989-1-AP, 1:1000) was purchased from Proteintech (Chicago, IL); anti-actin antibody (MAB1501, 1:10,000) was obtained from Millipore (Temecula, CA); and anti-αPEP7 (tyrosinase) antibody (1:1000) and anti-αPEP1 (TRP1) (1:1000) antibodies were kindly donated by V. J. Hearing (NIH, Bethesda, MD). For protein detection, the membranes were incubated with HRP-conjugated secondary antibodies and the signals were detected with SuperSignal^®^ West Dura HRP Detection Kit (Pierce, Rockford, IL).

### Immunofluorescence analysis by confocal microscopy

NHEMs, Melan-a, and B16F1 cells cultured on Lab-Tek^™^ glass chamber slides (Thermo Scientific, Waltham, MA) were washed with cold PBS, fixed for 20 min with 4% (w/v) paraformaldehyde, washed twice with PBS, and incubated in PBS containing 0.1% (v/v) Triton^™^ X-100 (PBS-T) for 3 min. The cells were further washed 3 times with PBS-T and then incubated with anti-polyglutamylated-tubulin antibody (1:100 dilution, Adipogene, AG-20B-0020), anti-acetylated-tubulin antibody (1:1000 dilution, Sigma-Aldrich), and anti-ARL13B antibody (1:200 dilution, Proteintech) diluted in PBS-T containing 5% (v/v) normal goat serum for 1 h at room temperature. After washing with PBS, the cells were incubated with appropriate conjugated secondary antibodies and then mounted on glass slides to observe fluorescence using a confocal laser scanning microscope (model LSM510; Carl Zeiss Microimaging Inc., Thornwood, NY).

### Statistical analysis

Data were obtained from at least 3 independent experiments and are presented as mean ± SE. Statistical evaluation of the results was performed using 1-way ANOVA (SPSS software, version 21).

## Result

### Primary cilium formation regulates melanogenesis in mouse melanocytes and melanoma cell lines

**Fig 1:**
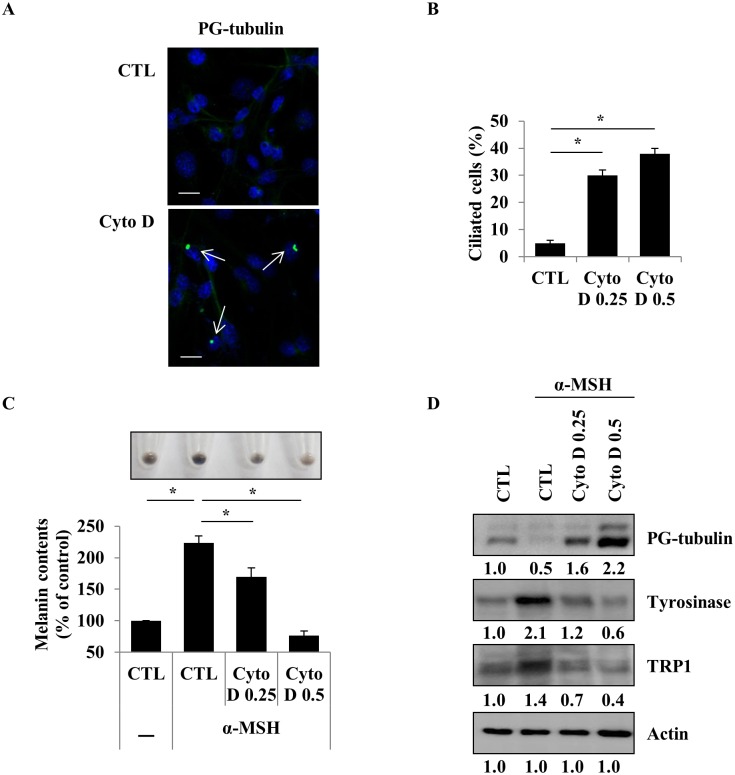
The induction of ciliogenesis reduces melanin synthesis in mouse normal melanocytes (Melan-a cells). Melan-a cells were treated with cytochalasin D (Cyto D, 0.25 μM) for 48 h. The induction of polyglutamylated-tubulin protein (PG-tubulin, green) was observed (A, scale bar = 10 μm). Ciliated cells were measured (Cyto D 0.25 and 0.5 μM, B). Melan-a cells pre-treated with α-MSH (1 μM) for 24 h were further treated with Cyto D (0.25 and 0.5 μM) for 48 h. Then, cellular melanin content was measured (C). The protein expression levels of PG-tubulin, tyrosinase, and TRP1 were analyzed (D). Data were obtained from at least 3 independent experiments and the values were presented as mean ± SE (* *p* < 0.05, ** *p* < 0.01).

Cilia are complex cellular organelles and they are composed of polarized structures. The both assembly (ciliogenesis) and disassembly of the cilium are interconnected with various cellular events, such as cell cycle, migration, polarization, vertebrate development, and genetic diseases [3]. Cytochalasin D (Cyto D), an actin polymerization inhibitor that causes cell cycle arrest at the G1-S transition, is wildly used as an inducer of primary cilium formation [27]. Previously, it was shown that the cilium is a tubulin-based cellular structure. Tubulin undergoes multiple post-translational modifications (PTMs). Among the PTMs of tubulin, polyglutamylation is thought to be associated with an active status of the tubulin in primary cilia [28]. Therefore, we analyzed the expression level of polyglutamylated tubulin protein to assess cilia formation. To assess the effect of Cyto D on cilium induction in mouse melanocytes, Melan-a cells were treated with Cyto D and the primary cilium formation was analyzed by confocal microscopy. In accordance with previous notion, treatment of Cyto D strongly increased the level of polyglutamylated tubulin and cilium assembly (Fig 1A and 1B). To further examine the change of melanin synthesis after cilium induction, the cells were treated with Cyto D and the cellular melanin contents were measured. α-MSH induced increase of melanin synthesis and decreased the protein expression level of polyglutamylated tubulin, but the α-MSH-induced increase of melanin synthesis was significantly reversed and the polyglutamylated tubulin level was significantly increased in Cyto D-treated Melan-a cells (Fig 1C and 1D). In addition, we further examined the expression levels of melanogenic enzymes in Cyto D-treated cells. Interestingly, the increased protein levels of tyrosinase and TRP1 by α-MSH treatment were significantly reduced by Cyto D in Melan-a cells (Fig 1D).

**Fig 2:**
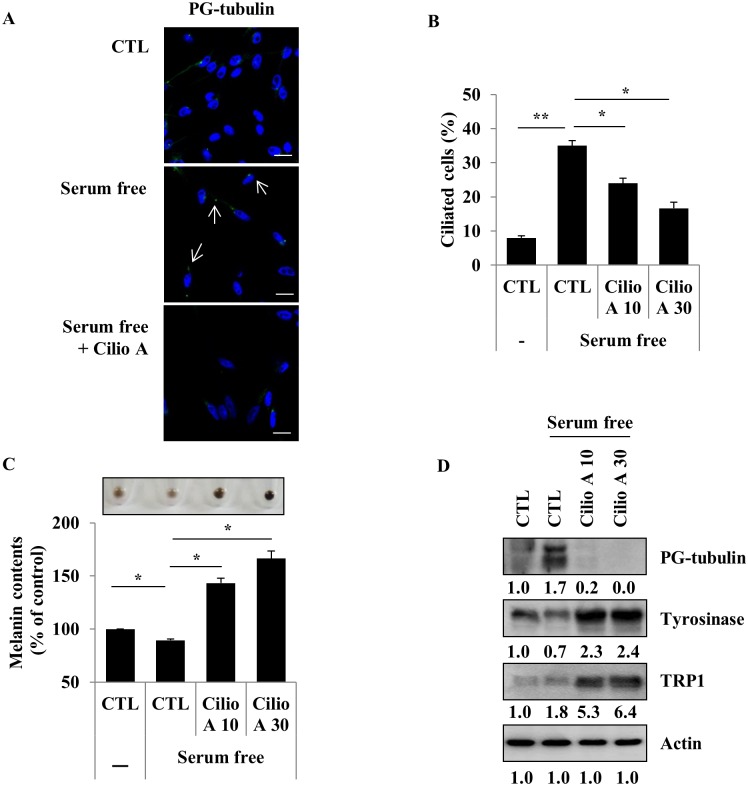
Inhibition of primary cilia induces melanin synthesis in Melan-a cells. Melan-a cells were incubated with serum free media and treated with ciliobrevin A1 (Cilio A, 10 μM) for 48 h. Primary cilia stained by polyglutamylated-tubulin antibody (green) were observed (A, scale bar = 10 μm) and ciliated cells were counted (Cilio A 10 and 30 μM, B). The cellular melanin content was measured (C). The expression levels of polyglutamylated-tubulin, tyrosinase, and TRP1 were analyzed (D). Data represent the mean ± SE of 3 independent experiments (* *p* < 0.05, ** *p* < 0.01).

Serum starvation was previously shown to induce primary cilium formation in different cell types, including htRPE (telomerase-immortalized human retinal pigmented epithelial) cells [27]. Therefore, we evaluated the effect of serum starvation on primary cilium formation in melanocytes. As shown in Fig 2, serum-free culture of the cells strongly induced primary cilium formation (Fig 2A and 2B). Ciliobrevin A1 (Cilio A), the first specific small-molecule antagonist for the cytoplasmic dyneins, was reported to inhibit primary cilium formation [29]. To examine the effect of Cilio A on cilium assembly in melanocytes, we treated melanocytes with Cilio A under serum-free conditions. Treatment with Cilio A significantly suppressed the serum starvation-induced primary cilium formation in Melan-a cells (Fig 2A and 2B). We further examined the changes of melanin synthesis and protein expression levels of melanogenic enzymes and polyglutamylated tubulin after the induction of primary cilium formation by serum starvation and its suppression by Cilio A treatment. Serum starvation slightly reduced the cellular melanin contents and tyrosinase and TRP1 protein expression levels, but treatment with Cilio A efficiently increased melanin synthesis and tyrosinase and TRP1 protein expression levels in Melan-a cells (Fig 2C and 2D). Polyglutamylated tubulin, which was increased by serum starvation, was dramatically reduced by Cilio A treatment in Melan-a cells (Fig 2D), suggesting that melanogenesis is modulated by primary cilia in melanocytes.

**Fig 3:**
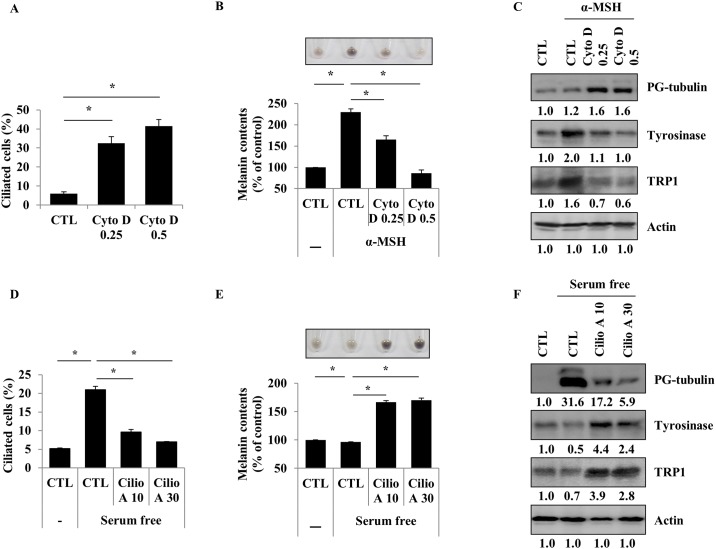
Ciliogenesis negatively regulates melanogenesis in B16F1 cells. B16F1 cells were treated with cytochalasin D (0.25 or 0.5 μM) for 48 h. The proportion of ciliated cells was measured (A). B16F1 cells were pre-treated with α-MSH (1 μM). After 24 h, they were treated with cytochalasin D (0.25 or 0.5 μM). After 48 h, cellular melanin content was measured (B). Polyglutamylated-tubulin, tyrosinase, and TRP1 were analyzed (C). B16F1 cells were treated with ciliobrevin A1 (10 or 30 μM) in serum-free media for 48 h. The proportion of ciliated cells was evaluated (D) and cellular melanin content was measured (E). Polyglutamylated-tubulin, tyrosinase, and TRP1 were analyzed (F). Data represent the mean ± SE of 3 experiments (* *p* < 0.05, ** *p* < 0.01).

These phenomena were confirmed in B16F1 mouse melanoma cell lines. Induction of ciliogenesis by Cyto D reduced α-MSH induced melanin synthesis in B16F1 cells (Fig 3A–3C). The reduction of ciliogenesis by Cilio A induced melanin synthesis and the protein expression of melanogenic enzymes in B16F1 cells (Fig 3D–3F). Taken together, these results suggested that primary cilia formation has an influence on melanogenesis in melanocytes and melanoma cell lines.

### Smo-GLI2 signaling, which is involved in ciliogenesis, regulates melanogenesis

**Fig 4:**
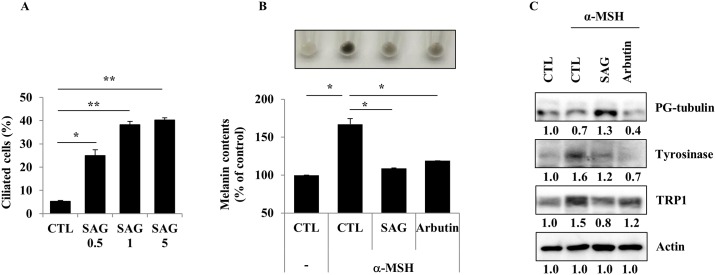
Activation of SHH signaling inhibits melanogenesis in B16F1 cells. B16F1 cells were treated with the ciliogenesis activator, Smo-agonist (SAG; 0.5 or 1 μM) for 24 h. The proportion of ciliated cells was measured (A). B16F1 cells were pre-treated with α-MSH (1 μM). After 24 h, the cells were further co-treated with α-MSH (1 μM) and SAG (5 μM) or arbutin (500 μM). After 24 h, the cellular melanin content was measured (B). The protein expression levels of tyrosinase and TRP1 were analyzed (C). Data represent the mean ± SE of 3 independent experiments (* *p* < 0.05, ** *p* < 0.01).

**Fig 5:**
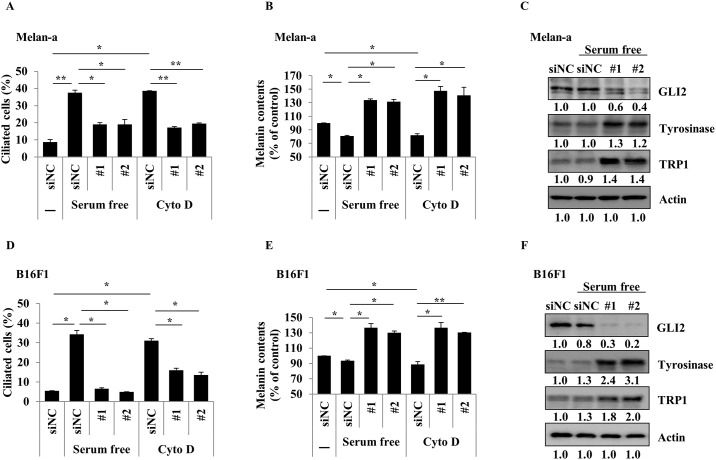
GLI2 mediates the regulation of melanogenesis by primary cilia. Melan-a cells were transfected with scrambled control (siNC) or GLI2 siRNA. After 24 h, the cells were treated with cytochalasin D (0.5 μM) or serum-free media. After 48 h, the proportion of ciliated cells was counted (A). Cellular melanin content was analyzed (B). GLI2, tyrosinase, and TRP1 were analyzed (C). B16F1 cells were transfected with siNC or GLI2 siRNA. After 1 day, the cells were treated with cytochalasin D (0.5 μM) or serum-free media for 2 days. Ciliated cells and cellular melanin content were analyzed (D, E). GLI2, tyrosinase, and TRP1 were analyzed (F). Data represent the mean ± SE of 3 experiments (* p < 0.05, ** p < 0.01).

Primary cilium formation is regulated by SHH and Wnt signaling pathways, but the major contributor to its induction is SHH signaling. SHH ligand activates the signaling pathway via the activation of Smo protein. The activated Smo protein enhances the activity of GLI transcription factors. Activated GLI transcription factors regulate the expression of genes such as those modulating renal patterning and cell cycle and those downstream of the SHH signaling pathway [10]. Notably, the MITF and GLI2 genes are inversely expressed in various melanoma cell lines [20]. Therefore, we investigated the involvement of Smo and the GLI2 transcription factor in the regulation of melanogenesis via primary cilia formation. B16F1 cells were treated with the Smo-agonist SAG (0.5 or 1 μM). After 24 h, an increased proportion of ciliated cells was observed (Fig 4A) and the induction of ciliogenesis by SAG led to the inhibition of melanogenesis in B16F1 cells cultured with α-MSH (Fig 4B). Consistently, the protein expression levels of tyrosinase and TRP1 were also decreased when ciliogenesis of the cells was activated by SAG (Fig 4C). Melan-a cells were transfected with either siRNA against GLI2 or scrambled control siRNA. After 48 h, the cells were exposed to Cyto D or serum-free conditions to induce ciliogenesis for 2 days. As shown in Fig 5, treatment of Cyto D and incubation in serum-free conditions strongly induced primary cilium formation. However, stimuli (Cyto D and serum starvation)-induced primary cilium formation was significantly suppressed in GLI2 knockdown cells (Fig 5A). We further assessed the cellular melanin content after the treatment of GLI2 knockdown cells with Cyto D and incubation in serum-free medium. Incubation in serum-free medium and treatment with Cyto D in control siRNA-treated cells slightly suppressed the cellular melanin content. However, the melanin content was significantly increased in GLI2 knockdown cells (Fig 5B). We next examined the expression of melanogenic enzymes in GLI2 knockdown cells. Genetic depletion of GLI2 increased the tyrosinase and TRP1 protein levels in Melan-a cells, compared with those in the wild-type cells (Fig 5C). These results suggested that the inhibition of melanogenesis by Cyto D or serum starvation was mediated through the induction of ciliogenesis via the Smo-GLI2 signaling pathway. In addition, we also found that serum starvation- and Cyto D-induced ciliogenesis was suppressed in GLI2 knockdown B16F1 mouse melanoma cells. The expression of melanogenic proteins and melanin content were increased in GLI2 knockdown B16F1 cells, similar to the results observed for Melan-a cells (Fig 5D–5F), implicating that Smo-GLI2-mediated signaling is involved in the regulation of melanin production by primary cilia.

### Ciliogenesis regulates melanogenesis in normal human epidermal melanocytes (NHEMs) and 3-dimensional human skin equivalents

**Fig 6:**
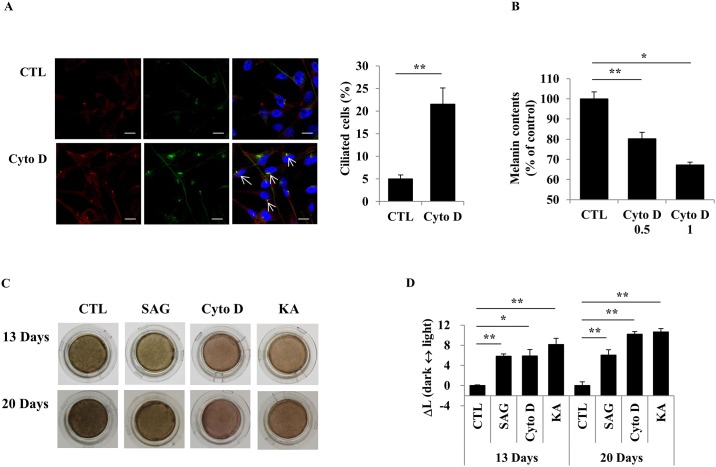
Induction of ciliogenesis suppresses pigmentation in normal human epidermal melanocytes (NHEMs) and a human skin model. NHEMs were treated with cytochalasin D (0.5 μM) for 48 h. After immunostaining for acetylated-tubulin (green) and ARL13B (red), ciliated cells were counted (A, scale bar = 10 μm). NHEMs treated with cytochalasin D (0.5 or 1 μM) for 48 h were harvested to measure the cellular melanin content (B). Photographs of 3-dimensional human skin substitutes treated with Smo-agonist SAG (5 μM), cytochalasin D (0.5 μM), or kojic acid (5 mM) for 13 and 20 days were taken (C). The pigmentation of the skin substitutes was measured and ΔL was calculated between the control and treated groups (D). Data represent the mean ± SE of 3 experiments (* p < 0.05, ** p < 0.01).

We further examined the regulation of melanogenesis via the ciliogenesis in NHEMs and 3-dimensional human skin equivalents (a reconstituted epidermis model containing normal human epidermal keratinocytes and NHEMs). NHEMs were treated with Cyto D for 2 days. Primary cilia in NHEMs were observed by immunostaining for acetylated tubulin (the active form of tubulin) and ARL13B (a small G protein localized in the cilia). Increase of primary cilia and decrease of melanin synthesis were induced by Cyto D treatment on NHEMs (Fig 6A and 6B). We further assessed the effect of ciliogenesis on skin pigmentation by administering the Smo agonist SAG and Cyto D to 3-dimensional human skin equivalents for 13 and 20 days. Skin pigmentation was reduced by activators of primary cilia formation in the human skin model (Fig 6C and 6D). Taken together, these results further suggested that ciliogenesis also regulates melanogenesis in NHEMs and pigmentation in 3-dimensional human skin equivalents.

## Discussion

Through the cilium, cells and tissues adapt to changes in the extracellular environment by regulating cell cycle, cellular function, cell shape, and movement. For the cilium to work as an antenna, a variety of receptors, transporter proteins, ion channels, and effector molecules are localized to the cilium [2]. Signaling pathways orchestrated by primary cilia include signaling through Ca^2+^, SHH, Wnt, and signal transduction via the ECM [30]. When the SHH signaling pathway is activated, the GLI2 and Smo proteins translocate from the cell body into the primary cilia. In this report, we tested the role of primary cilia on melanogenesis in melanoma cells and melanocytes. Increased melanin synthesis of the cells exposed to α-MSH was significantly reduced when the cells were treated with Cyto D, a primary cilium formation inducer (Figs 1 and 3). Under serum-free conditions, primary cilia formation was elevated, while melanogenesis was slightly down-regulated. However, the suppression of melanogenesis induced by serum starvation was fully rescued and melanogenesis was significantly further elevated in comparison with the control cells by treatment with Cilio A, an inhibitor of primary cilia formation (Figs 2 and 3). Melanosomes are transported along microtubules from the perinuclear region to dendrites by the motor proteins kinesin and dynein. Subsequently, melanosomes move along actin filaments on the inner aspect of the plasma membrane of dendrites and are transferred into neighboring keratinocytes. If melanosome transport and transfer are inhibited, melanin content is also decreased [31]. Cyto D is an actin polymerization inhibitor and Cilio A is an antagonist of the cytoplasmic dyneins. Therefore, Cyto D and Cilio A might be also able to inhibit melanogenesis by disturbing melanosome movement on intermediate and actin filaments independently of primary cilia. However, Cilio A was found to induce melanin synthesis of the cells. Moreover, inhibition of melanin synthesis in the pigment cells by the induction of primary cilia formation by Cyto D treatment or serum starvation was recovered by treatment with GLI2 siRNA (Fig 5). GLI2 is an important transcription factor for primary cilia formation and signaling through primary cilia. The Smo agonist SAG also inhibited melanin synthesis in melanocytes and pigmentation in a human skin model (Fig 6). Therefore, our study suggested that a connection exists between primary cilia and melanogenesis, in that primary cilia are required for the regulation of melanogenesis. In skin, exposure to UVR in sunlight induced α-MSH secretion from keratinocytes and then α-MSH, in turn, increases the cAMP level in melanocytes. cAMP then activates its downstream molecules PKA and CREB protein 1. Activation of these proteins up-regulates the expression of MITF. cAMP was reported to inhibit the SHH signaling pathway and GLI2 expression [32]. GLI2 is a critical transcription factor that antagonizes MITF expression [20]. Therefore, UVR increases skin pigmentation by elevating cAMP signaling, which leads to inhibition of the antagonizing effect of GLI2 on MITF, although further study is necessary to confirm that MITF is a direct target of GLI2. It is possible that GLI2 acts separately from primary cilia to regulate melanogenesis. However, the localization of GLI2 on the primary cilia is crucial for its transcriptional activity [33]. Consequently, these data demonstrated that primary cilia regulate melanogenesis via signaling involving Smo-GLI2.

The primary cilia play an important role during development [34]. Therefore, the function of the primary cilia is probably still important in the stem cell populations of various adult tissues. Adult keratinocyte stem cells exist on the basement membrane between the epidermis and dermis of skin. From there, stem cells stratify, differentiate, and generate the mature epidermal cells. Hair follicle (HF) morphogenesis is also initiated from the stem cells by signal crosstalk between epithelial and mesenchymal cells. If primary cilia are eliminated from the embryonic epidermis or cultured keratinocytes, cell differentiation is inhibited and hyperproliferation of the cells is induced [22]. HF morphogenesis is also impaired by the elimination of the primary cilia because signaling crosstalk between epithelial and mesenchymal cells that is critical for HF growth and maturation is mediated by primary cilia. However, in this study, we showed that melanogenesis, which is the final stage of melanocytic differentiation, was inversely proportional to ciliogenesis. This distinctive feature of melanocytes may be attributed to their different degree of differentiation, because melanocytes always make melanin at a basal level and then the melanogenesis is elevated by external stimuli such as UVR. Therefore, it seems likely that primary cilia in melanocytes are necessary for maintenance of the sub-differentiated state and for quick response to external stimuli. After external stimuli are sensed by primary cilia, melanogenesis is increased and ciliogenesis is decreased.

Future studies on the molecular mechanism underlying ciliogenesis in melanocytes may need to investigate whether cilia have a role in the pathogenesis of hyper- or hypo-pigmentation disorders of the human skin and hair. Nevertheless, our results suggest that a potential agent for the treatment of undesirable skin pigmentation disorders can be developed from lead compounds that regulate ciliogenesis.
